# A Prospective Observational Study of Ventral Hernia

**DOI:** 10.7759/cureus.28240

**Published:** 2022-08-21

**Authors:** Gauri S Jadhav, Geet R Adhikari, Rajashree S Purohit

**Affiliations:** 1 Department of General Surgery, Dr. Hedgewar Hospital, Aurangabad, IND; 2 Department of General Surgery, Om Multispecialty Hospital, Malkapur, IND

**Keywords:** complications of ventral hernia, ventral hernia presentation, epigastric hernia, umbilical hernia, incisional hernia, prospective observational study, clinical study, ventral hernia

## Abstract

Background

Ventral hernias are commonly encountered problems in the field of general surgery. Incisional hernia is a common complication following abdominal surgery that requires reoperation. This study was conducted to understand the incidence of various types of ventral hernia in both sexes and various age groups, predisposing factors, clinical features, and complications.

Methods

This prospective observational study was conducted in the Department of Surgery, Dr. Hedgewar Hospital, Aurangabad, Maharashtra, India, on a total of 100 patients diagnosed with anterior abdominal wall hernia between September 2020 to February 2021. Data collection included thorough history taking and clinical examination along with relevant investigations. The data collected was entered in a proforma, tabulated, and analyzed with the IBM SPSS Statistics for Windows, Version 24.0 (Released 2016; IBM Corp., Armonk, New York, United States).

Results

Incisional hernia (43%) was the most common of all ventral hernias. Females were much more affected than males. Out of these types studied, epigastric hernia showed more incidence among males. The average age of presentation was 52 years. Obesity was the most common predisposing factor (34%) with female preponderance. Obese patients were also associated with a higher rate of postoperative complications like wound infection and seroma. In the cases of incisional hernia, 32.6% of the patients gave a history of previous surgery complicated by a wound infection. Incisional hernias were more common in lower midline incisions (34.9%) and after gynecological surgery (55.81%) like total abdominal hysterectomy, cesarean section, or tubal ligation. In the majority of the patients (62.8%), the incisional hernia occurred within three years of the previous surgery. Wound infection following ventral hernia repair occurred in 11% of the cases, wound dehiscence in 3%, and seroma in 2 % of the cases.

Conclusions

The most common ventral hernias in decreasing order of their frequency are incisional hernia, umbilical hernia, para-umbilical hernia, and epigastric hernia. Epigastric and umbilical hernias are more common in males whereas incisional hernia is more common in females. Obesity and constipation were found to be the major predisposing risk factors. Incisional hernia is more common in females after gynecological and obstetrics surgery. The lower midline segment is the most common site for developing an incisional hernia.

## Introduction

The word hernia is derived from the Latin word meaning rupture. It occurs when an organ that is normally contained in a body cavity protrudes through the lining of that cavity [1]. A ventral hernia is defined as a protrusion through the anterior abdominal wall fascia. These defects on the anterior abdominal wall fascia can be categorized into spontaneous (or primary) or acquired (incisional). They can also be categorized by their location on the abdominal wall. An epigastric hernia occurs from the xiphoid process to the umbilicus, an umbilical hernia occurs at the umbilicus, and the hypogastric hernia occurs below the umbilicus. An acquired hernia occurs on a previously operated site and hence is termed an incisional hernia [2]. In October 2008, the European Hernia Society (EHS) introduced an elaborated classification of ventral hernias [3].

Ventral hernias may or may not be symptomatic. They usually present as a swelling over the abdomen associated with or without pain and rarely with complications like strangulation or incarceration. The incisional hernia is a common long-term complication of abdominal surgeries and the incidence ranges from 2-20% [4,5]. The overall incidence of incisional hernia is slightly higher in the midline laparotomy incision as compared to the transverse incision [5].

An understanding of the abdominal wall anatomy and physiology is key to the restoration of abdominal wall function. Prevention of incisional hernia still requires evaluation, but proper closure of laparotomy and abdominal incisions, in general, using the correct technique, and correct suture material may reduce the incidence. Preventing wound infection is vital in preventing incisional hernia in the future. The operative management of ventral hernias constitutes a wide range of surgeries from classical anatomical repair of the defect, prosthetic repair, and advanced reconstruction methods. In addition to this, the use of minimally invasive procedures allows for less surgical site infection, shorter hospital stay, and an early return to work.

This study was undertaken to understand the demographics and clinical profile of various types of ventral hernias in the population visiting a tertiary care health center in India.

## Materials and methods

A prospective observational study was conducted in the Department of Surgery, Dr. Hedgewar Hospital, Aurangabad, India on a sample size of 100 patients diagnosed with anterior abdominal wall hernia between September 2020 to February 2021. The sample size calculated on the statistician’s opinion was 93, which was rounded to 100. The institutional ethics committee, Dr. Babasaheb Ambedkar Medical Research Society Ethics Committee, Dr. Hedgewar Hospital, approved this study in December 2019 (approval number ECR-641/MH/2019) for 18 months. The cases were thoroughly examined and investigated to be considered appropriate to be a part of this study. Outpatient clinical notes, discharge summary, operative notes, and laboratory data were reviewed.

All uncomplicated cases of ventral hernia were surgically managed with open onlay mesh repair using a synthetic polypropylene (prolene) mesh. In the technique, the hernia defect was primarily closed, a polypropylene mesh was placed over the anterior rectus sheath and fixed with prolene 2-0 round body suture. Complicated cases with strangulation or risk of contamination were managed with anatomical repair only (without mesh placement). Patients were followed up at regular intervals for six months to assess the outcomes and post-operative complications. The data collected was entered in proforma, tabulated, and analyzed with IBM SPSS Statistics for Windows, Version 24.0 (Released 2016; IBM Corp., Armonk, New York, United States).

Patients aged less than 18 years, those with femoral hernias, inguinal hernias, posterior abdominal wall hernias, and those who did not wish to undergo open surgery were excluded from this study.

## Results

In the present study out of 100 cases of ventral hernia, 43 were diagnosed with an incisional hernia (43%), which was the most common variety. Twenty-nine patients had an umbilical hernia (29%), 18 had a paraumbilical hernia (18%), and 10 patients had an epigastric hernia (10%). Majority of the patients with a ventral hernia in this study presented in the sixth decade of life with the average age of presentation being 52 years. The mean age of presentation in this study was 62 years for epigastric hernia, 50 years for umbilical hernia, and 55 years for paraumbilical hernia and incisional hernia.

Ventral hernias were overall more common in women. Out of the 100 patients in the study, 59% were females and 41% were males. Epigastric hernias were more common in males (70%), while incisional hernias were more common in females (76%).

**Figure 1:**
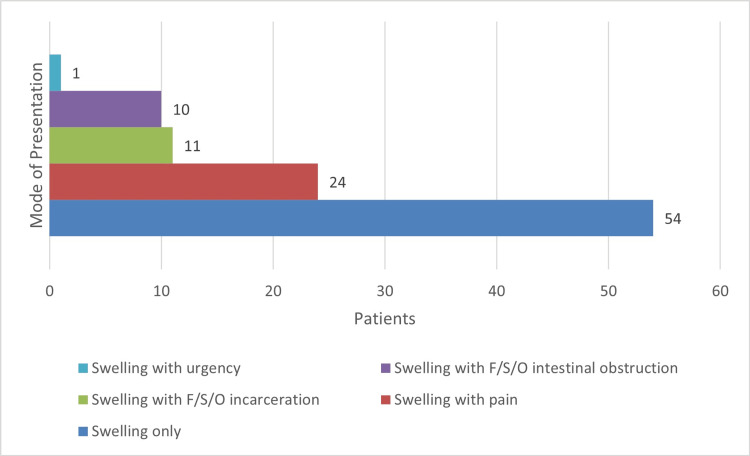
Mode of presentation of ventral hernia F/S/O: Features suggestive of

Out of the 100 cases, 54 (54%) presented with the chief complaint of swelling only, 24 cases (24%) presented with swelling and pain, 11 (11%) cases presented with the features of incarceration, and 10 (10%) presented with the features of intestinal obstruction. One case in our study had an interesting mode of presentation of swelling with urinary urgency (Figure 1).

Many patients had co-morbidities that were seen either alone or in combination with others. Of these, 4% had only diabetes mellitus, 12% suffered from hypertension, 10% patients had diabetes along with hypertension, and 1% had chronic obstructive pulmonary disease (COPD). One patient had diabetes, hypertension, and COPD.

Multiple risk factors such as obesity, history of chronic cough, heavy weight lifting, constipation, benign hyperplasia of the prostate (BHP), and smoking were found to be associated in the present study with varying degrees, out of which obesity was found to be the most common association in 34% of the patients. Constipation was also one of the major risk factors seen in 24% of the patients interfering with wound healing and increasing the risk of the development of incisional hernia in such patients.

The hernia defect size was measured on preoperative ultrasound in all the patients. This gap measured up to 2 cm in 45% of patients. In 28% of patients, it was 2-4 cm, and in 27% of patients, it was found to be more than 4 cm.

**Figure 2:**
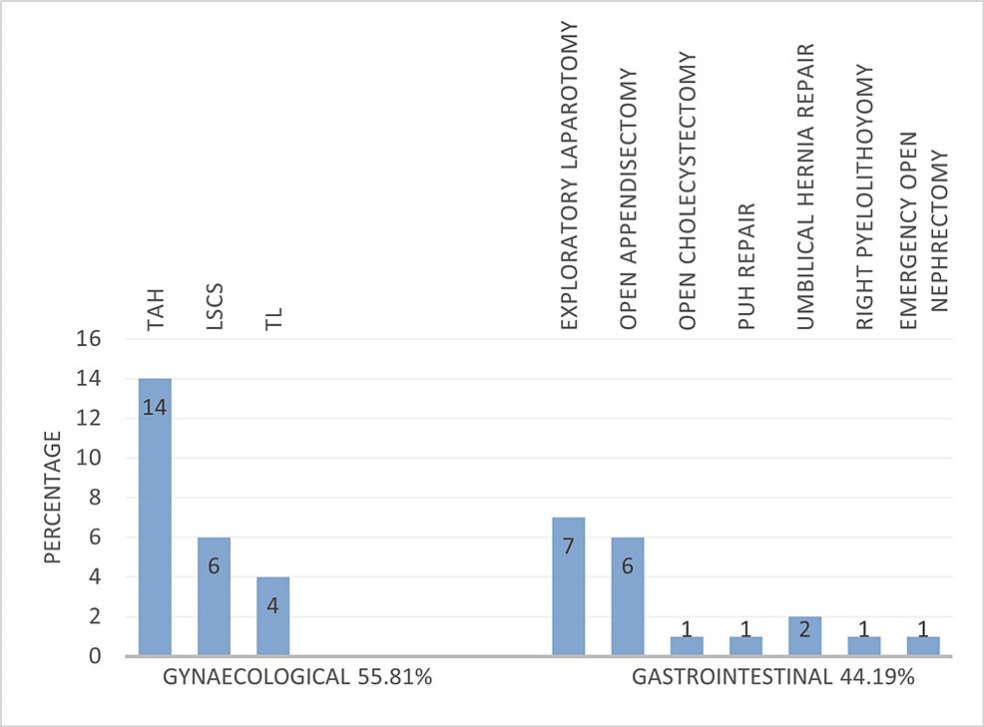
Percentage distribution of previous surgeries leading to incisional hernia TAH: Total abdominal hysterectomy; LSCS: Lower segment cesarean section; TL: Tubal ligation, PUH: Paraumbilical hernia

Of the patients presenting with an incisional hernia, 55.81% reported a history of gynecological surgery in the past out of which, a majority of them underwent total abdominal hysterectomy (TAH) followed by lower section cesarean section (LSCS) and tubal ligation (TL). Of the patients with an incisional hernia, 44.19% reported a history of gastrointestinal surgery in the past out of which, a majority of them underwent exploratory laparotomy (Figure 2).

The type of incision taken in previous surgery was observed in the cases of incisional hernia. A lower midline incision scar was observed in 15 out of 43 cases (34.9%) whereas a transverse incision scar was found in 28% of the cases. Other incisions observed were oblique, paramedian, and upper midline. Moreover, 14 patients (32.6%) with incisional hernia reported a history of wound infection during the previous surgery.

**Figure 3:**
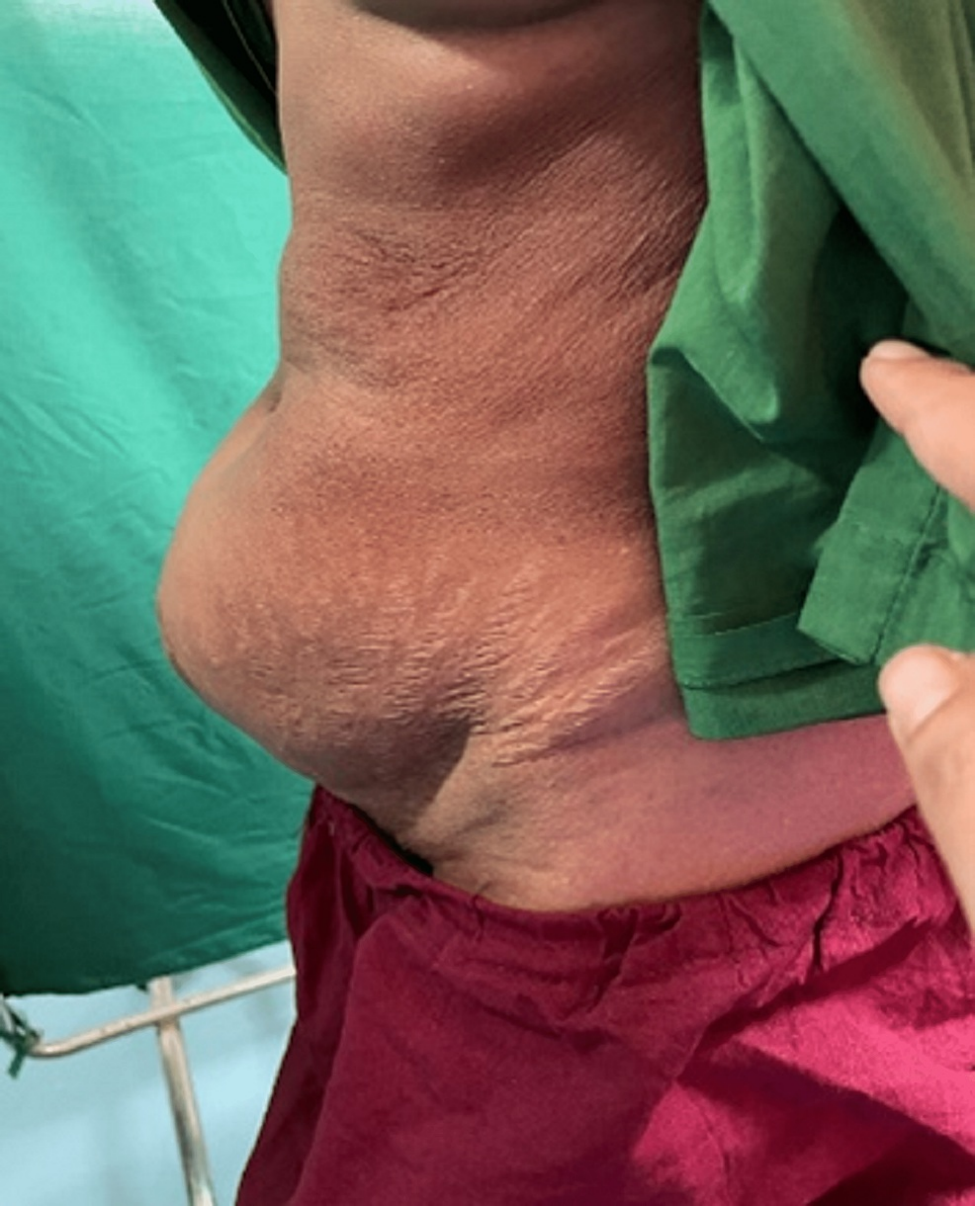
A clinical image of infra-umbilical incisional hernia

The most common anatomical site for ventral hernia was the infra-umbilical incisional variety (Figure 3) with 38% cases followed by umbilical (29%), paraumbilical (18%), and epigastric (10%); supraumbilical incisional variety is the least common (5%) (Table 1).

**Table 1:** Distribution of anatomical site for ventral hernia

ANATOMICAL SITE	PERCENTAGE
Incisional Hernia-Infraumbilical (IH-IU)	38
Incisional Hernia-Supraumbilical (IH-SU)	5
Umbilical (U)	29
Paraumbilical (PUH)	18
Epigastric (E)	10
TOTAL	100

The contents of the sac in 49% of the patients were omentum, in 32% it was bowel, and in 18% it was both omentum and bowel. One case had a urinary bladder as the content of the sac. Of the cases, 10% presented with abdominal swelling along with the clinical features of intestinal obstruction, of which 3% had strangulated bowel loop requiring resection and anastomosis with the anatomical repair of the anterior abdominal wall without mesh placement.

In this study, 84% had no postoperative complications. 11% developed wound infection, 2% developed seroma requiring aspiration, and 3% developed wound dehiscence requiring secondary suturing. There was no surgery-related mortality in this study during a postoperative follow-up period of six months.

## Discussion

The results of the present study have been compared to other series of similar nature. In the present study, it was found that incisional hernia was the most common variety of ventral hernia (46%). Bose et al. showed in their study that incisional hernia was the most common variety (62.8%) [6]. In our study, the mean age of presentation was 52 years. It was maximum for epigastric hernia (62 years) followed by incisional hernia (55 years). Epigastric hernia and umbilical hernia showed a male preponderance with 70% and 52% cases, respectively. These findings coincided with the study conducted by Courtney et al. [7]. Of patients with incisional hernias, 77% were females. Elderly patients have an invariable association with several comorbid conditions, which increases the risk of intra-operative and postoperative complications favoring the formation of incisional hernia. Overall, ventral hernias are more common in females with a male-to-female ratio of 1:1.4 approximately. Multiple factors such as multiparity, decreased abdominal muscle tone, replacement of collagen tissues, history of gynecological surgeries through a lower midline incision, etc., predispose females to ventral hernias [5,8].

**Figure 4:**
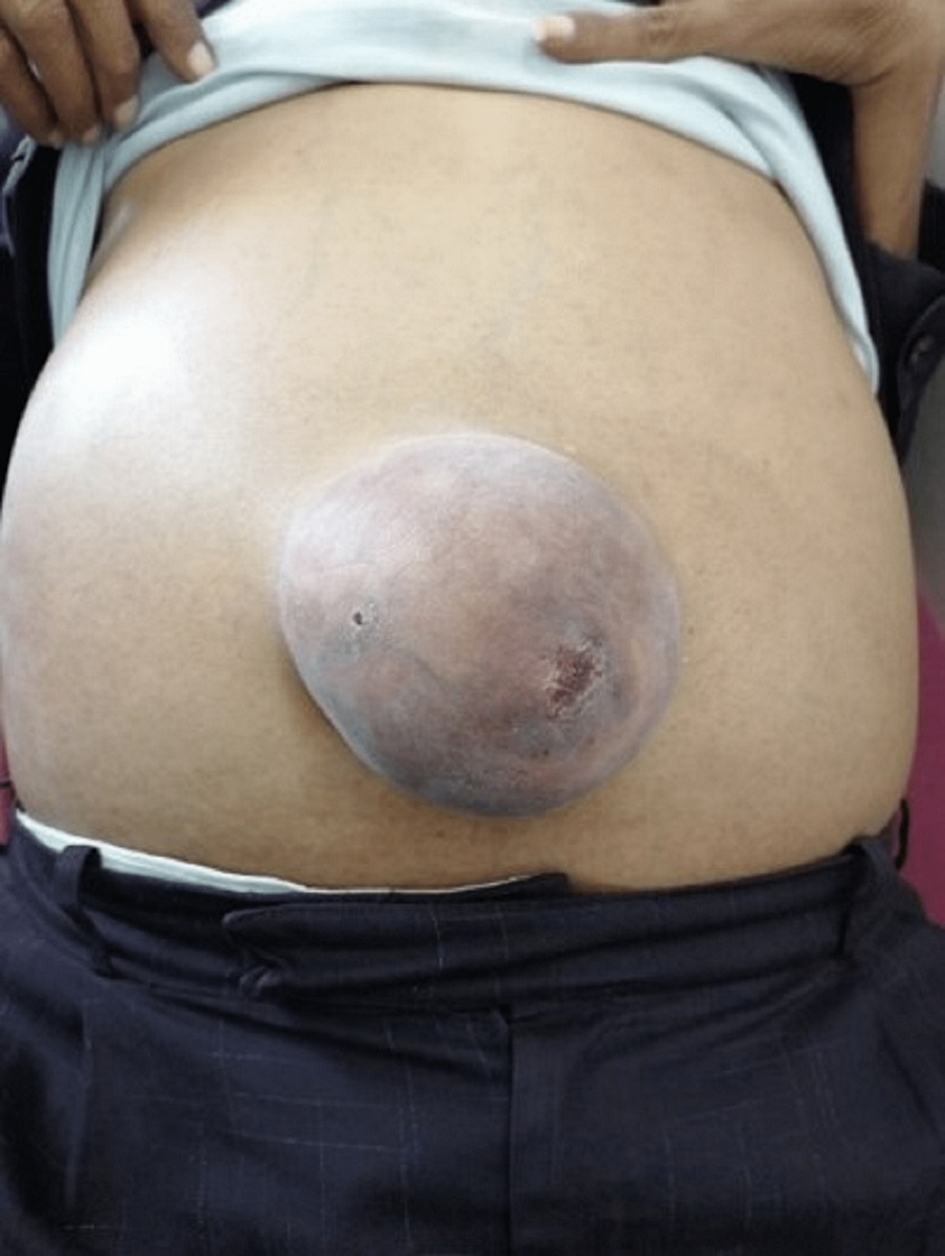
Clinical image of obstructed umbilical hernia

The most common mode of presentation in this study was abdominal swelling alone (54%) followed by swelling with pain (24%), swelling with irreducibility (11%), and swelling with features of intestinal obstruction (10%) (Figure 4). One case presented with swelling and urinary urgency. These findings were consistent with that in a previous study by Jaykar et al. [9].

Diabetes mellitus, obesity, and smoking have been associated with a high percentage of postoperative hernias [10]. Several pathogenic mechanisms seem to be involved. Peripheral tissue hypoxia, reduced collagen type I to type III ratio, and degradation of connective tissue caused by an imbalance between proteases and their inhibitors play a vital role [11,12]. Gecim et al. studied 109 cases of incisional hernia and concluded that chronic constipation was one of the most common factors associated with late recurrence [13].

In our study, 34% of patients were obese, 24% had a history of chronic constipation, 14% had a history of lifting heavy weights, 10 % had a chronic cough, 10% suffered from benign prostatic hyperplasia (BPH), and 6% were chronic smokers. The findings were comparable to a study done by Jaykar et al., where 16% were obese, 34% had chronic constipation, 16% were smokers, and 12% had BHP [9].

About 55.81% of cases of incisional hernia in our study occurred following gynecological procedures. A higher incidence of incisional hernia was seen in lower midline incisions (34.9%). This is comparable with the studies done by Shukla et al. (53%), Goel et al. (44.6%), and Parekh et al. (51%) [14-16]. It could be attributed to the increased intra-abdominal pressure in the lower abdomen during the erect posture and the absence of a posterior rectus sheath below the arcuate line [14]. In a study conducted by Toms et al., it was concluded that incisional hernias are more common following midline incision through the relatively avascular line and are less common following transverse incision, especially where muscle splitting approaches have been used [17].

The size of the defect in the abdominal wall was noted preoperatively along with the contents of the sac by ultrasonography and was later confirmed by intra-operative findings. The hernia was classified as small, medium, or large based on the size of the defect according to the European hernia society guidelines [18]. A majority of the patients (45%) had a defect size of less than 2 cm as measured in preoperative radiological studies.

Amongst the cases of incisional hernia in this study, the surgical wound infection rate following previous surgery was found to be 32.6%. Various studies have shown that the incisional hernia becomes evident within the first year of surgery but the patient may present with the hernia swelling as late as 10 years or even more [19]. Our results were comparable to a previous study done by Jaykar et al. [9]. Ten cases presented with swelling along with features of intestinal obstruction, three of them needed resection and anastomosis due to bowel strangulation followed by anatomical repair due to the risk of potential contamination. The rest of the cases were treated with onlay mesh repair using a polypropylene mesh.

**Figure 5:**
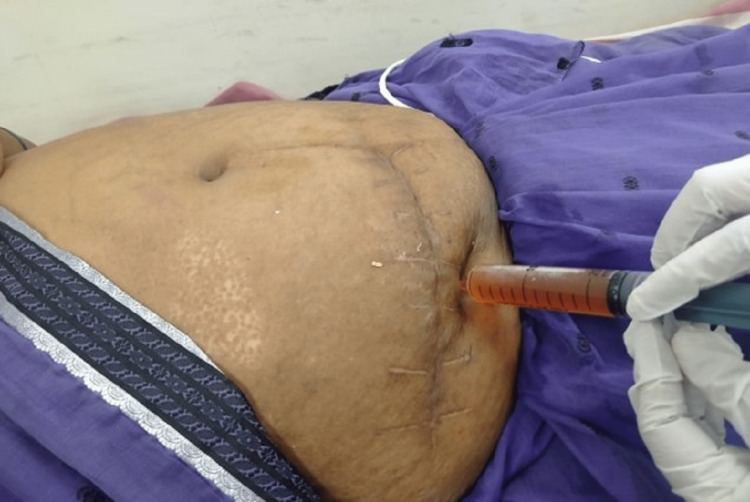
A clinical image showing aspiration of seroma developed in a postoperative patient of an incisional hernia repair, which was managed conservatively by aspiration and pressure dressing

The duration of surgery for onlay mesh hernioplasty was 96.65 minutes in our study. It is comparable to 83.41 minutes in a similar previous study done by Alsounday et al. [20]. In the present study, it was found that 2% of cases developed postoperative seroma (Figure 5), 11% had surgical wound infection, and 3% had wound dehiscence. The overall postoperative local complication rate was found to be 16%. This postoperative wound infection rate was comparable to the previous studies based on onlay mesh repair for ventral hernia done by Alsounday et al. and Godara et al., where the infection rate was found to be to be 15% [20,21].

Limitations of the study

The data collection was confined to a particular limited area of the country, which may not be an accurate presentation of the general population. The sample size was very small; hence, it represents only a small proportion of the entire population in the country. In view of a very limed period of follow-up and small sample size, it was not possible to comment on recurrence rates. We recommend a large multicentric trial with a longer duration of follow-up to measure the recurrence of the condition.

## Conclusions

The most common ventral hernias in decreasing order of their frequency are incisional hernia, umbilical hernia, paraumbilical hernia, and epigastric hernia respectively. Epigastric and umbilical hernias are more common in males whereas incisional hernia is more common in females. Obesity and constipation are observed to be the major predisposing risk factors. Incisional hernia is more common after obstetric and gynecological surgery like total abdominal hysterectomy, cesarean section, or tubal ligation. The lower midline segment is the more common site for developing an incisional hernia. The presence of wound infection in a previous surgery predisposes the patient to the development of an incisional hernia. 
